# Adult-onset hypothyroidism induces granulosa cell apoptosis and affects ovarian follicle development in rats

**DOI:** 10.3389/fcell.2025.1610694

**Published:** 2025-05-30

**Authors:** Shuyue Li, Lina Zhang, Wanqiong Li, Jiaying Qin, Lingbin Qi, Xi Xiao, Zhigang Xue, Jinfeng Xue, Yazhong Ji

**Affiliations:** ^1^ Reproductive Medical Center, Department of Obstetrics and Gynecology, Tongji Hospital, Tongji University School of Medicine, Shanghai, China; ^2^ Stem Cell Research Center, School of Medicine, Tongji University, Shanghai, China; ^3^ Translational Center for Stem Cell Research, Tongji Hospital, Tongji University School of Medicine, Shanghai, China

**Keywords:** adult-onset hypothyroidism, granulosa cell, apoptosis, oocyte, oxidative stress

## Abstract

**Introduction:**

Hypothyroidism is a common endocrine disorder in women, which could lead to ovulation disorders and infertility, however, the effects of adult-onset hypothyroidism on ovarian development and gene expression characteristics need further study.

**Methods:**

Here we conducted an adult-onset hypothyroidism rat model by using the methimazole (MMI) induction, then the hormone level changes and ovarian development were evaluated, furthermore, the effects of gene expression of granulosa cells and oocytes were detected by using single-cell RNA sequencing.

**Results:**

Our results showed that, in addition to a decrease in thyroid hormones, the body weight was significantly reduced, while the estrus cycle was prolonged in the hypothyroidism group. Although the ovary/body weight ratio was not changed, the adult-onset hypothyroidism disrupted follicle development, primarily manifested by an increased number of atretic follicles and a decreased number of corpora lutea. Serum sex hormone levels were also imbalanced, with elevated LH, FSH, and PRL, while E2 and P were decreased. By combining single-cell RNA sequencing and the validation experiments, we found that adult-onset hypothyroidism promoted apoptosis in granulosa cells of antral follicles and induced oxidative stress in oocytes. Notably, we found significant heterogeneity in mitochondrial ROS in the control group, indicating differences in the redox status of different normal oocytes, which disappeared after hypothyroidism promoted oxidative stress.

**Discussion:**

In conclusion, adult-onset hypothyroidism interferes with normal follicle development and impairs fertility by promoting apoptosis in granulosa cells of antral follicles and inducing oxidative stress in oocytes.

## Introduction

Hypothyroidism is a common endocrine disorder in women of childbearing age which could lead to the infertility (de Oliveira et al., 2022; Meng et al., 2016; Li and Chan, 2020; Feldt-Rasmussen et al., 2024). The prevalence of overt hypothyroidism in women of childbearing age ranges from 0.2% to 4.5%, while subclinical hypothyroidism is 5%–7% (Poppe, 2021). Compared to men, women are more prone to developing hypothyroidism (Li et al., 2020). Previous studies have shown that hypothyroidism can also lead to menstrual irregularities, ovulation disorders, affect embryo implantation and development (Brown et al., 2023; Chen et al., 2023).

The ovary is the female reproductive organ, responsible for producing mature oocytes (Gershon and Dekel, 2020). The normal development of ovarian follicles is fundamental to achieving these functions. Under physiological conditions, it requires regulation by the hypothalamic-pituitary-ovarian (HPO) axis (Koysombat et al., 2024). The hypothalamus secretes gonadotropin-releasing hormone (GnRH), which acts on the pituitary gland. In response, the pituitary gland secretes gonadotropins that promote follicle development and the synthesis of sex hormones, such as E2 and P (Chen X. et al., 2022). Abnormalities of the HPO axis can lead to disorders in follicle development (Physiopathological determinants of human infertility, 2002; Mikhael et al., 2019). Meanwhile, ovarian theca cells, granulosa cells, and oocytes all express thyroid hormone receptors (THRs). Therefore thyroid hormones can directly regulate follicle development through THRs (Zheng et al., 2015). Previous studies have shown that hypothyroidism can reduce the number of antral follicles and increase the number of atretic follicles in rats, possibly related to oxidative stress or nitric oxide synthase system dysfunction (Meng et al., 2016; Xu et al., 2020; Wang et al., 2021a). However, the effects of hypothyroidism on follicle development, especially in the oocytes and granulosa cells, have not yet been well explored.

In the present study, we utilized a rat model to investigate how adult-onset hypothyroidism affects follicle development. We found that adult-onset hypothyroidism can lead to ovarian dysfunction in rats, affecting the synthesis of sex hormones and ovulation, which may be caused by the increase apoptosis of granulosa cells in antral follicles and induce oxidative stress in oocytes.

## Materials and methods

### Experimental design

The Animal Experiment Administration Committee of Tongji University approved all animal experimental procedures (TJAA06422201). Thirty-five female Sprague-Dawley (SD) rats, aged 10–12 weeks (weighing 220–270 g), were housed in the specific pathogen-free (SPF) facility of Tongji Hospital with free access to commercial chow. The rats were randomly assigned to control and hypothyroidism (hypo) groups. The hypo group (20 rats) received drinking water containing 0.02% (w/v) methimazole (MMI) for 4 weeks (Kent et al., 2022), while the control group (15 rats) received drinking water. During the modeling period, the estrus cycle of the rats was monitored daily using vaginal smears, and their body weight was measured weekly.

After 4 weeks, thyroid tissue and serum were collected. Hypothyroidism was mainly evaluated by serum levels of thyroid-stimulating hormone (TSH), free triiodothyronine (fT3), and free thyroxine (fT4). The serum levels of sex hormones were also measured. The bilateral ovaries were weighed and subjected to morphological evaluations. Superovulation was induced to assess ovulation capacity. Oocytes and cumulus granulosa cells were collected, and single-cell libraries were established using the Smart-seq2 method for high-throughput sequencing.

### Enzyme-linked immunosorbent assay (ELISA)

Blood was collected through cardiac puncture and left at room temperature for 1 h. It was then centrifuged at 1,000 *g* for 20 min at 4°C to obtain serum. According to the manufacturer’s instructions, serum levels of thyroid hormones and sex hormones were measured using an ELISA kit (Bim, USA). Sex hormones include follicle-stimulating hormone (FSH), luteinizing hormone (LH), estradiol (E2), progesterone (P), and prolactin (PRL). Each group included at least three biological samples, each with at least three technical replicates.

### Hematoxylin and eosin (H&E) staining

The thyroids and ovaries were quickly collected, with fat removed, and fixed in 4% paraformaldehyde. After dehydration with ethanol, the tissues were embedded in paraffin. Serial sections of the embedded tissue were made, each with a thickness of 5 μm (Chen M. et al., 2022). For ovarian tissue, 10 sections were randomly selected from the largest cross-section for H&E staining, and the number of follicles at different stages was counted. The criteria for determining the stages of follicles have been described previously (Fu et al., 2017). Ovarian follicles are classified into primordial follicles (PrF), primary follicles (PF), secondary follicles (SF), antral follicles (AF), corpora lutea (CL), and atretic follicles (AtF) (Zhou et al., 2023).

### Superovulation

After completing the modeling, superovulation was performed in both rat groups. 40 IU of pregnant mare serum gonadotropin (PMSG) (Sansheng, China) was administered via intraperitoneal injection, followed by 40 IU of human chorionic gonadotropin (hCG) (Sansheng, China) 48 h later. After 14–16 h, cumulus-oocyte complexes (COCs) were collected from the ampulla of the fallopian tubes (Wang et al., 2020). COCs were then placed in a solution of hyaluronidase (Aibei, China) for 2–3 min to obtain denuded oocytes. The number of oocytes obtained and the proportion of abnormally morphologic oocytes were recorded.

### Construction of cDNA library and single-cell RNA sequencing

Oocytes and cumulus granulosa cells were collected through superovulation, with the control group collecting 39 oocytes and their corresponding cumulus granulosa cells, and the hypo group collecting 35. The cDNA library was constructed using the Smart-seq2 method. In brief, cells were lysed to extract mRNA. The mRNA was then reverse transcribed into cDNA, followed by amplification and purification. The cDNA was fragmented, adapters were added and then amplified and purified to prepare the library. Sequencing was performed using the Illumina NovaSeq 6000 system.

We utilized the FPKM value as the index for measuring gene expression and analyzed gene expression in the two groups. Differentially expressed genes (DEGs) of the cumulus granulosa cells or oocytes between the hypo group and control group were identified based on | log_2_ (fold change) | >1 and *P* value <0.05 using the R package. Gene ontology (GO) enrichment analysis was conducted using the R topGO package and the DAVID online tool (https://david.ncifcrf.gov/).

### Quantitative real-time PCR (qPCR)

Ovaries were collected on the day of estrus and placed in pre-cooled M2 medium (Aibei, China). 26-gauge needles were used to puncture the antral follicles to release granulosa cells. The granulosa cells were collected from the medium by centrifugation. Following the manufacturer’s instructions, mRNA was extracted using TRIzol (Invitrogen, USA). Reverse transcription was performed using the RevertAid First Strand cDNA Synthesis Kit (Thermo Scientific, USA), and qPCR was completed using TB Green *Premix Ex Taq* II (Takara, Japan). mRNA levels were normalized to *β-actin.* Each experiment was repeated at least three times independently. The primer sequences are shown in Supplementary Table S1.

### Terminal deoxynucleotidyl transferase (TdT)-mediated dUTP nick end labeling (TUNEL) staining

Ovarian paraffin sections were stained using the TUNEL staining kit (elabscience, China). In brief, sections were deparaffinized, hydrated, and permeabilized. The sections were then incubated with the Labeling Solution. DNA was subsequently stained with DAPI. After staining, the sections were observed using the fluorescence microscope (Nikon, Eclipse Ti).

### Fluorescence staining

Oocytes were incubated in M2 medium containing 10 μM H2DCFDA (MCE, USA), 5 μM MitoSOX Red (MCE, USA), and 10 μg/mL Hoechst 33342 (Beyotime, China) at 37°C in a 5% CO_2_ atmosphere for 15 min. They were then washed three times in the M2 medium and observed under the fluorescence microscope.

### Statistical analysis

All data were analyzed using GraphPad Prism 8 and presented as mean ± standard error of the mean (SEM). Each experiment was repeated at least three times. If the data conformed to the normal distribution, the t-test was used. If not, the Mann-Whitney test was used. *P* value <0.05 was considered a significant difference. Significance was defined as *P < 0.05, **P < 0.01, ***P < 0.001 and ****p < 0.0001.

## Results

### Prolonged estrus cycle in rats induced by adult-onset hypothyroidism

**FIGURE 1:**
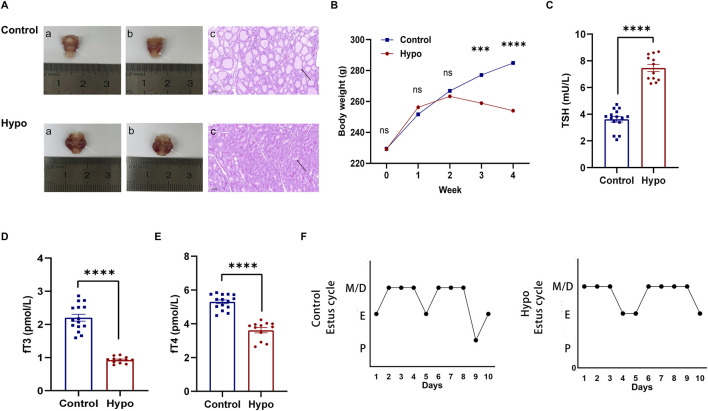
Establishment of hypothyroidism and evaluation of estrus cycle. **(A)** Morphological evaluation of the thyroid in the control and hypo groups. (white arrow: thyroid, black arrow: thyroid follicle). **(B)** Body weight changes in the control group and hypo groups during modeling (n = 15 for control group, n = 20 for hypo group), ^ns^ *p* > 0.05, ***P < 0.001, ****p < 0.0001. **(C–E)** Serum levels of TSH, fT3, and fT4 in the control and hypo groups. Data are presented as Mean ± SEM (n = 5 for the control group, n = 4 for the hypo group), ****p < 0.0001. **(F)** Representative images of the estrus cycle in the control and hypo groups.

To investigate the effect of adult-onset hypothyroidism on the ovarian follicle development, we first established a rat model by using methimazole (MMI). After for 4 weeks inducation, we found the thyroids were significantly enlarged in the hypothyroidism (hypo) group, while H&E staining showed that the volume of thyroids follicles were significantly smaller than that in the control group, and the colloid in the follicular cavities were reduced (Figure 1A), while the body weight of the hypo group were significantly lower than that of the control group and showed a decreasing trend starting from the third week (Figure 1B). The hormone assay showed the level of TSH in the hypo group was significantly higher compared with the control group, while the levels of fT3 and fT4 were remarkably lower (Figures 1C–E; Supplementary Table S2), which was consistent with the hormonal changes of hypothyroidism. These results suggested that the model was successfully established (Korkmaz et al., 2023).

To determine the effect of hypothyroidism on the estrus cycle, we detected the vaginal smears for each phase including proestrus (P), estrus (E), metestrus (M), and diestrus (D) (Salleh et al., 2022; Guillén-Ruiz et al., 2021) (Supplementary Figure S1A), the results showed that the estrus cycle of rats in the hypo group became irregular, primarily manifested as a significantly prolonged estrus cycle, extended from 4.37 ± 0.18 days to 6.48 ± 0.21 days, while the proportion of proestrus in the overall estrus cycle was decreased from 9.37% ± 1.88%–5.43% ± 1.69%, and the proportion of estrus tended to increase (Table 1; Supplementary Figures S1B,C). These data suggested that the estrus cycle was abnormal in rats with adult-onset hypothyroidism. The Figure 1F reflects the typical estrus pattern of the two groups of rats.

**TABLE 1:** Duration of estrus cycle and proportion of each estrus stage in control and hypo group.

Characteristics	Control group (n = 15)	Hypo group (n = 20)	*P* value
Estrus cycle (d)	4.37 ± 0.18	6.48 ± 0.21	0.0000
P (%)	9.37 ± 1.88	5.43 ± 1.69	0.0447
E (%)	33.33 ± 2.79	37.53 ± 2.49	0.2710
M/D (%)	57.30 ± 3.80	57.04 ± 2.34	0.5696

Data are presented as Mean ± SEM (n = 15 for the control group, n = 20 for the hypo group).

### Abnormal ovarian follicle development in rats with adult-onset hypothyroidism

**FIGURE 2:**
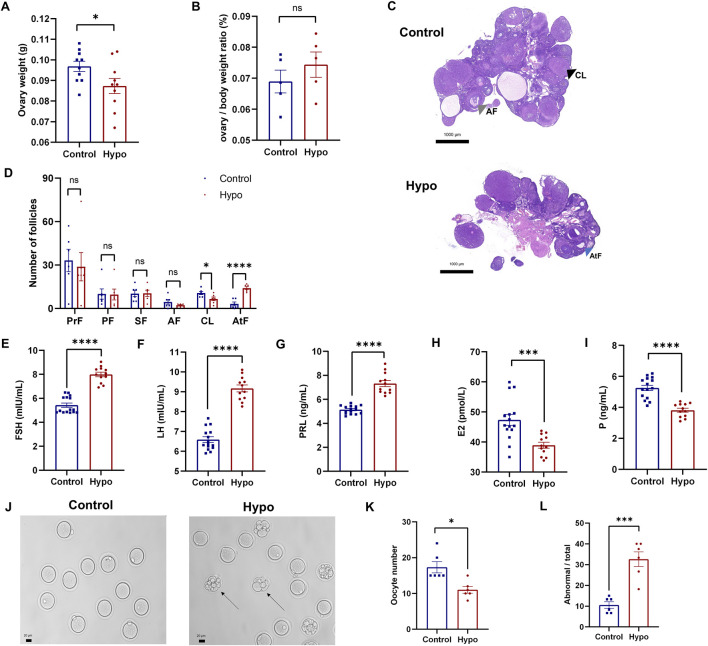
Hypothyroidism leads to abnormal development of ovarian follicles in rats. **(A)** Ovary weight in the control and hypo groups after modeling. Data are presented as Mean ± SEM (n = 5 for each group), *p < 0.05. **(B)** Ovary-to-body weight ratio in the control and hypo groups after modeling. Data are presented as Mean ± SEM (n = 5 for each group), ^ns^ *p* > 0.05. **(C,D)** Representative images of H&E staining of the ovaries (right panel, grey arrow: AF, black arrow: CL, blue arrow: AtF) and statistical analysis of the number of follicles at different stages (left panel). Data are presented as Mean ± SEM (n = 3 for each group, 5 sections per ovary are assessed), ^ns^ *p* > 0.05, *p < 0.05, ****p < 0.0001. **(E–I)** Serum levels of FSH, LH, PRL, E2, and P in rats. Data are presented as Mean ± SEM (n = 5 for the control group, n = 4 for the hypo group), ***P < 0.001, ****p < 0.0001. **(J–L)** Representative images of oocytes (left, black arrow: oocytes with abnormal morphology) and quantitative analysis of the number (midddle) and proportion of abnormal morphology (right) in the control and hypo groups. Data are presented as Mean ± SEM (n = 6 for each group), *p < 0.05, ***p < 0.001.

To evaluate the impact of hypothyroidism on follicular development, we first collected and weighed the ovaries from both groups. Although the ovarian weight in the hypo group was significantly lower (Figure 2A), there was no significant difference in the ovary-to-body weight ratio between the two groups (Figure 2B). Furthermore, H&E staining showed that the number of corpora lutea (CL) in the hypo group was significantly reduced, and the number of atretic follicles (AtF) was remarkably increased, with a trend towards a decrease in antral follicles (AF) (Figures 2C,D). Additionally, serum sex hormone levels showed significant abnormalities. Compared to the control group, levels of FSH, LH, and PRL significantly increased, while levels of E2 and P significantly decreased in the hypo group (Figures 2E–I; Supplementary Table S3). These results confirmed the effects of hypothyroidism on serum sex hormone levels and follicular development.

Furthermore, we assessed ovulation in both groups of rats (Figures 2J–L). Compared to the control group, the number of oocytes retrieved from the hypo group was significantly lower, while the proportion of abnormally morphologic oocytes increased (Figures 2K,L), indicating that hypothyroidism led to abnormal ovarian follicle development and negatively impacts ovulation.

### Adult-onset hypothyroidism induced apoptosis in granulosa cells of antral follicles

**FIGURE 3:**
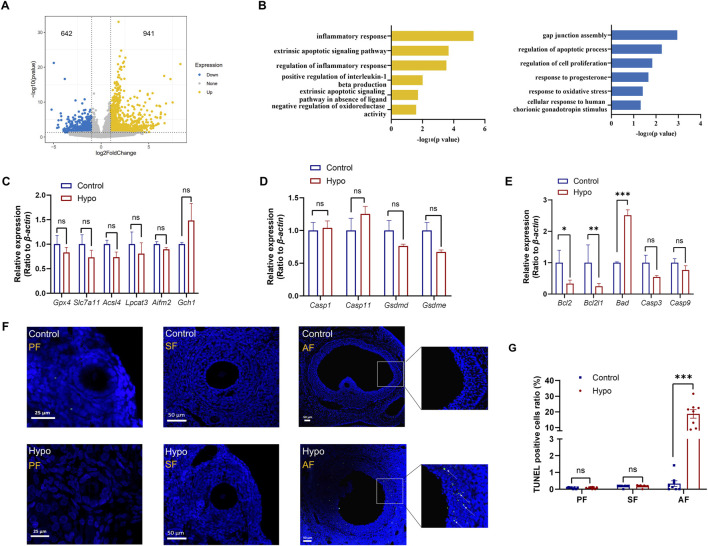
Adult-onset hypothyroidism induces apoptosis in granulosa cells of antral follicles. **(A)** Volcano plot of differentially expressed genes (DEGs) in granulosa cells of the hypo group, with 941 upregulated and 642 downregulated genes. (n = 5 for control group, n = 4 for hypo group) **(B)** Plot illustrating the GO enrichment analysis of DEGs in granulosa cells of the hypo group. **(C,D,E)** Relative gene expression levels of ferroptosis (left), pyroptosis (middle), apoptosis (right) in control and hypo groups. Data are presented as Mean ± SEM (n = 4 for each group), ^ns^ *p* > 0.05, *p < 0.05, **p < 0.01, ***p < 0.001. **(F)** TUNEL staining of follicles at different stages in both the control and hypo groups. (n = 3 for each group, 3 sections per ovary are assessed, white arrow: TUNEL-positive) **(G)** Quantitative analysis of TUNEL staining in two groups. Data are presented as Mean ± SEM (n = 3 for each group), ^ns^ *p* > 0.05, ***p < 0.001.

Granulosa cells provide material support and facilitate signal transduction for oocytes during follicle development (Chen et al., 2021; Li et al., 2022). To further investigate how hypothyroidism impairs ovulation capacity, we focused on the potential changes in granulosa cells and oocytes. After collecting oocytes and corresponding cumulus granulosa cells from the control and hypo groups, the transcriptome was detected by using single-cell RNA sequencing. Compared to those in the control group, granulosa cells in the hypo group had a total of 1,583 differentially expressed genes (DEGs), with 941 upregulated and 642 downregulated (Figure 3A). GO enrichment analysis showed that the upregulated DEGs were mainly enriched in apoptosis, inflammation, and oxidative stress, while the downregulated DEGs were primarily involved in gap junctions, cell proliferation, and sex hormone response (Figure 3B), suggesting that the damage to granulosa cell function, including increased apoptosis and decreased proliferation ability, may be a critical cause of follicular atresia and oocyte maturation disorders.

Apoptosis is one of the common ways of cell death. Single-cell RNA sequencing data suggest that adult-onset can promote granulosa cell apoptosis. In addition, previous studies have shown that ferroptosis and pyroptosis are also associated with granulosa cell death in polycystic ovarian syndrome (PCOS) and diminished ovarian reserve (DOR) (Wang et al., 2025; Yang et al., 2025), therefore, we investigated three major cell death pathways: ferroptosis, pyroptosis, and apoptosis. The ferroptosis-related genes, including Glutathione Peroxidase 4 (*Gpx4*), Solute Carrier Family 7 Member 11 (*Slc7a11*), Acyl-CoA Synthetase Long Chain Family Member 4 (*Acsl4*), Lysophosphatidylcholine Acyltransferase 3 (*Lpcat3*)*,* Apoptosis Inducing Factor Mitochondria Associated 2 (*Aifm2*), and GTP Cyclohydrolase 1 (*Gch1*), and the pyroptosis-related genes, including Caspase 1 (*Casp1*), Caspase 11 (*Casp11*)*,* Gasdermin D (*Gsdmd*), and Gasdermin E (*Gsdme*), were analyzed by qPCR. The results showed no significant differences in the expression levels of these genes between the control and hypo groups (Figures 3C,D), indicating that the death of granulosa cells in the hypo group is not due to ferroptosis or pyroptosis.

We also detected the key genes involved in the apoptosis pathway, including BCL2 Apoptosis Regulator (*Bcl2*), BCL2 Like 1 (*Bcl2l1*), BCL2 Associated Agonist Of Cell Death (*Bad*), Caspase 3 (*Casp3*), and Caspase 9 (*Casp9*). The results indicated that the expression levels of *Bcl2* and *Bcl2l1* were significantly decreased in the hypo group, while the expression level of *Bad* was remarkably increased. No significant differences were observed in the expression levels of *Casp3* and *Casp9* between the groups (Figure 3E). These results are consistent with scRNA-seq data, suggesting an enhancement of the apoptotic pathway in granulosa cells. Furthermore, we conducted TUNEL staining on paraffin sections of ovaries from both groups, focusing on the apoptosis status of granulosa cells. The results showed no significant differences in the proportion of TUNEL-positive granulosa cells between the groups in primary and secondary follicles. However, in the hypo group, especially in the pre-ovulatory stage of antral follicles (AF), the proportion of TUNEL-positive granulosa cells was significantly increased (Figures 3F,G). These results suggested that adult-onset hypothyroidism has no effect on granulosa cells in the primary and secondary stages, but significantly induced apoptosis at the antral follicle stage, which may impact oocyte development.

### Adult-onset hypothyroidism leads to oxidative stress in oocytes

**FIGURE 4:**
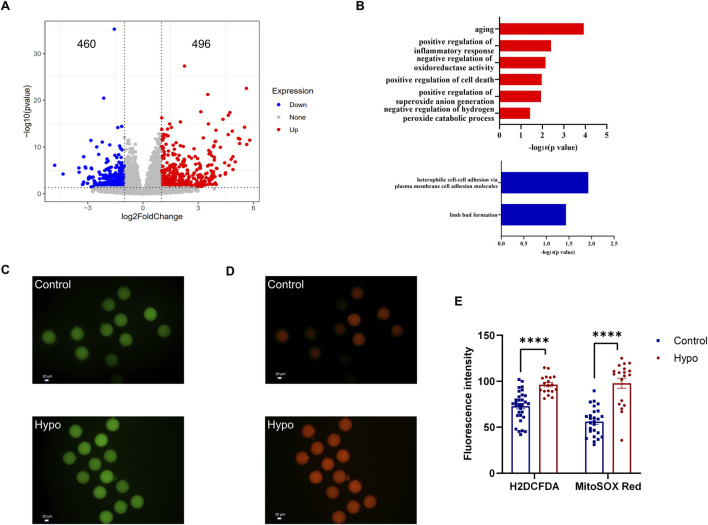
Adult-onset hypothyroidism leads to oxidative stress in oocytes. **(A)** Volcano plot of DEGs in oocytes of the hypo group, indicating 496 upregulated and 460 downregulated genes. (n = 5 for the control group, n = 4 for the hypo group) **(B)** Plot illustrating the GO enrichment analysis of DEGs in oocytes of the hypo group. **(C,D)** Representative images of H2DCFDA (left) and MitoSOX RED (right) staining in oocytes. (n = 6 for each group). **(E)** Quantitative analysis of fluorescent staining intensity between the two groups. Data are presented as Mean ± SEM (n = 6 for each group), ****p < 0.0001.

To further investigate the effects of hypothyroidism and granulosa cell apoptosis on oocytes, we further analyzed single-cell transcriptome data. The analysis revealed a total of 956 DEGs in the hypo group, with 496 genes upregulated and 460 genes downregulated (Figure 4A). GO enrichment analysis indicated that the upregulated DEGs in the hypo group were mainly enriched in pathways related to oxidative stress, cell death, and aging, while the downregulated DEGs were enriched in cell adhesion and subsequent embryo development (Figure 4B). These findings suggest that excessive cell death and oxidative stress were common characteristics between granulosa cells and oocytes in hypothyroidism.

During the maturation of oocytes, the dynamic balance between intracellular oxidative and antioxidative systems must be maintained. Granulosa cells play a crucial role in maintaining the redox balance of oocytes, and an increase in granulosa cell apoptosis may lead to oxidative stress in oocytes (Shi et al., 2023). Sequencing results indicated that many upregulated DEGs in the hypo group were enriched in biological processes related to oxidative stress. Based on this, we conducted tests on relevant markers, which primarily included intracellular ROS levels (indicated by H2DCFDA staining, Green) and mitochondrial superoxide levels (indicated by MitoSOX Red staining, Red). Compared to the control group, the levels of intracellular ROS and mitochondrial superoxide were all significantly elevated in the hypo group (Figures 4C–E), indicating oxidative stress in oocytes of the hypo group. Notably, we found that the intracellular ROS levels of different oocytes in the control group were similar, but the levels of mitochondrial superoxide varied significantly, suggesting heterogeneity in mitochondrial superoxide levels among oocytes. However, mitochondrial superoxide levels in oocytes were increased and became similar in the hypo group (Figures 4C,D), likely due to the apoptosis of granulosa cells.

## Discussion

The effect of hypothyroidism on follicle development is urgent to explore due to its high incidence rate among women of childbearing age. In this study, we investigated the impact and potential mechanisms of adult-onset hypothyroidism on ovarian function using the MMI-induced rat model. We found that adult-onset hypothyroidism interfered with the normal development of follicles, leading to an increase in the number of atretic follicles. Single-cell RNA sequencing data and experimental results indicated that adult-onset hypothyroidism increased granulosa cell apoptosis, particularly in the pre-ovulatory stage of antral follicles. Such changes disrupted the microenvironment of oocyte growth, exacerbated oxidative stress, and led to a decrease in both the quantity and quality of oocytes, ultimately resulting in impaired fertility.

The hypo group showed significantly increased TSH levels and decreased fT3 and fT4 levels compared to the control group. When the body is in a hypothyroid state, the serum levels of fT3 and fT4 decrease (McDermott, 2020). Due to the negative feedback mechanism of the hypothalamic-pituitary-thyroid axis, the pituitary gland increases the secretion of TSH (Hershman and Beck-Peccoz, 2023). Therefore, the results of the serum thyroid hormone measurements suggested that we successfully established an adult-onset hypothyroidism model in female rats.

During the modeling process, we monitored the estrus cycles of the rats daily. As modeling progressed, the estrus cycles of the hypo group became irregular, characterized by prolonged cycles and a decreased proportion of the proestrus phase. Regular estrus cycles indicate sexual maturation in rats, primarily associated with the cyclical changes in sex hormones (Sadowska et al., 2022). Since the cyclicity of sex hormones is linked to follicular development, these irregular estrus cycles indirectly demonstrate abnormal follicular development in hypothyroid rats.

Compared to the control group, the hypo group exhibited abnormal follicular development, specifically characterized by an increased number of atretic follicles, and a reduced number of corpora lutea. Follicles are crucial for sex hormone synthesis. de Oliveira et al. (2022) showed that hypothyroidism can lead to a decrease in LH and an increase in P. However, our results indicated that the hypo group had lower levels of E2 and P, along with higher levels of FSH, LH, and PRL. E2, one of the most important female sex hormones, is primarily synthesized and secreted by the ovaries. Combined with ovarian H&E staining results, we hypothesized that the decrease in E2 was mainly associated with the increased number of atretic follicles. The reduced number of corpora lutea likely contributes to the decrease in P levels. Follicular development is regulated by the HPO axis (Sirotkin et al., 2024). The decrease in E2 and P levels triggers the pituitary to secrete more FSH and LH through a negative feedback mechanism, resulting in abnormally high levels of these hormones. Additionally, excessive secretion of PRL is primarily due to elevated levels of thyrotropin-releasing hormone (TRH) (Molitch, 1992). Increased PRL levels interfere with the pulsatile secretion of GnRH from the hypothalamus, further disrupting the function of the HPO axis (Szukiewicz, 2024).

We assessed the ovulation capacity of the two groups of rats, focusing on both the quantity and quality of oocytes as reported (Miao et al., 2020). Following methods reported in previous studies (Wang et al., 2020), we performed superovulation in the rats. The results revealed a significant decrease in the number of oocytes obtained from the hypo group, along with an increase in the proportion of oocytes with abnormal morphology. This indicated the reduction in both the quantity and quality of oocytes in the hypo group. In rats, the number of oocytes released is positively correlated with the number of offspring, while oocyte quality determines the developmental potential of the zygote (Suzuki et al., 2024). These findings suggested that adult-onset hypothyroidism negatively affects ovulation capacity.

Granulosa cells play a crucial role in follicle development (Chakravarthi et al., 2021), and their excessive death is a primary cause of follicular atresia. Previous studies have shown that inflammation and oxidative stress can promote ferroptosis or pyroptosis of granulosa cells (Wang et al., 2025; Yang et al., 2025). According to our single-cell RNA sequencing results, we found that many DEGs were enriched in pathways related to inflammation, oxidative stress and apoptosis. Based on this, we further investigated the mechanisms underlying the abnormal death of granulosa cells. The qPCR results demonstrated an enhanced apoptotic pathway in granulosa cells of the hypo group. Additionally, TUNEL staining of the ovary confirmed that this pathological process predominantly occurs at the antral follicle stage. During ovulation, appropriate granulosa cell apoptosis facilitates the rupture of follicle walls and the release of oocytes (Zhang et al., 2024). Therefore, excessive apoptosis of granulosa cells in antral follicles may be a significant contributor to follicular atresia in hypothyroid rats.

Under physiological conditions, the body’s oxidative and antioxidative systems maintain a dynamic balance. However, when oxidative substances increase abnormally or the body’s antioxidative capacity diminishes, oxidative products can accumulate, leading to oxidative stress (Wang et al., 2021b; Hussain et al., 2021). ROS accumulation is a major cause of oxidative stress (Chaudhary et al., 2023), which can result in oocyte aging, characterized by decreased quantity and quality (Wang et al., 2021b).

Based on our sequencing data, we examined indicators related to oocyte oxidative stress. Fluorescent staining of oocytes revealed significantly increased levels of ROS and mitochondrial superoxide in the hypo group, indicating the presence of oxidative stress. Granulosa cells play a crucial role in supporting oocyte development by providing essential materials such as glycolytic products, cholesterol, and amino acids (Liu et al., 2023). The increased apoptosis of granulosa cells in the antral follicles of the hypo group may disrupt the microenvironment necessary for normal oocyte development, thus contributing to oxidative stress. However, the detailed mechanisms behind these changes warrant further exploration.

In conclusion, this study utilized a rat model to investigate the effects of adult-onset hypothyroidism on ovarian follicle development. The pathological process of hypothyroidism induced granulosa cell apoptosis in antral follicles, leading to oxidative stress in oocytes. This disruption in follicular development ultimately impaired the fertility of female rats. Our findings underscore that hypothyroidism in adulthood significantly affects reproductive function by compromising follicle development.

## Data Availability

The datasets used and analyzed during the current study are available from the corresponding author on reasonable request.

## Glossary

HPO: hypothalamic-pituitary-ovarian
GnRH: gonadotropin-releasing hormone
THRs: thyroid hormone receptors
TSH: thyroid-stimulating hormone
fT3: free triiodothyronine
fT4: free thyroxine
FSH: follicle-stimulating hormone
LH: luteinizing hormone
E2: estradiol
P: progesterone
PRL: prolactin
PrF: primordial follicles
PF: primary follicles
SF: secondary follicles
AF: antral follicles
CL: corpora lutea
AtF: atretic follicles
PMSG: pregnant mare serum gonadotropin
hCG: human chorionic gonadotropin
COCs: cumulus-oocyte complexes
PCOS: polycystic ovarian syndrome
DOR: diminished ovarian reserve
DEGs: Differentially expressed genes
GO: Gene ontology
MMI: methimazole
TUNEL: Terminal deoxynucleotidyl transferase (TdT)-mediated dUTP nick end labeling
Gpx4: Glutathione Peroxidase 4
Slc7a11: Solute Carrier Family 7 Member 11
Acsl4: Acyl-CoA Synthetase Long Chain Family Member 4
Lpcat3: Lysophosphatidylcholine Acyltransferase 3
Aifm2: Apoptosis Inducing Factor Mitochondria Associated 2
Gch1: GTP Cyclohydrolase 1
Casp1: pyroptosis-related genes, including Caspase 1
Casp11: Caspase 11
Gsdmd: Gasdermin D
Gsdme: Gasdermin E
Bcl2: BCL2 Apoptosis Regulator
Bcl2l1: BCL2 Like 1
Bad: BCL2 Associated Agonist Of Cell Death
Casp3: Caspase 3
Casp9: Caspase 9
