# Supplementary material for: Adult-onset hypothyroidism induces granulosa cell apoptosis and affects ovarian follicle development in rats
Source: Front Cell Dev Biol. 2025 May 30;13:1610694. doi: 10.3389/fcell.2025.1610694 (PMC12163021; doi:10.3389/fcell.2025.1610694)
Supplement: Supplementary file 1 [file DataSheet1.docx]

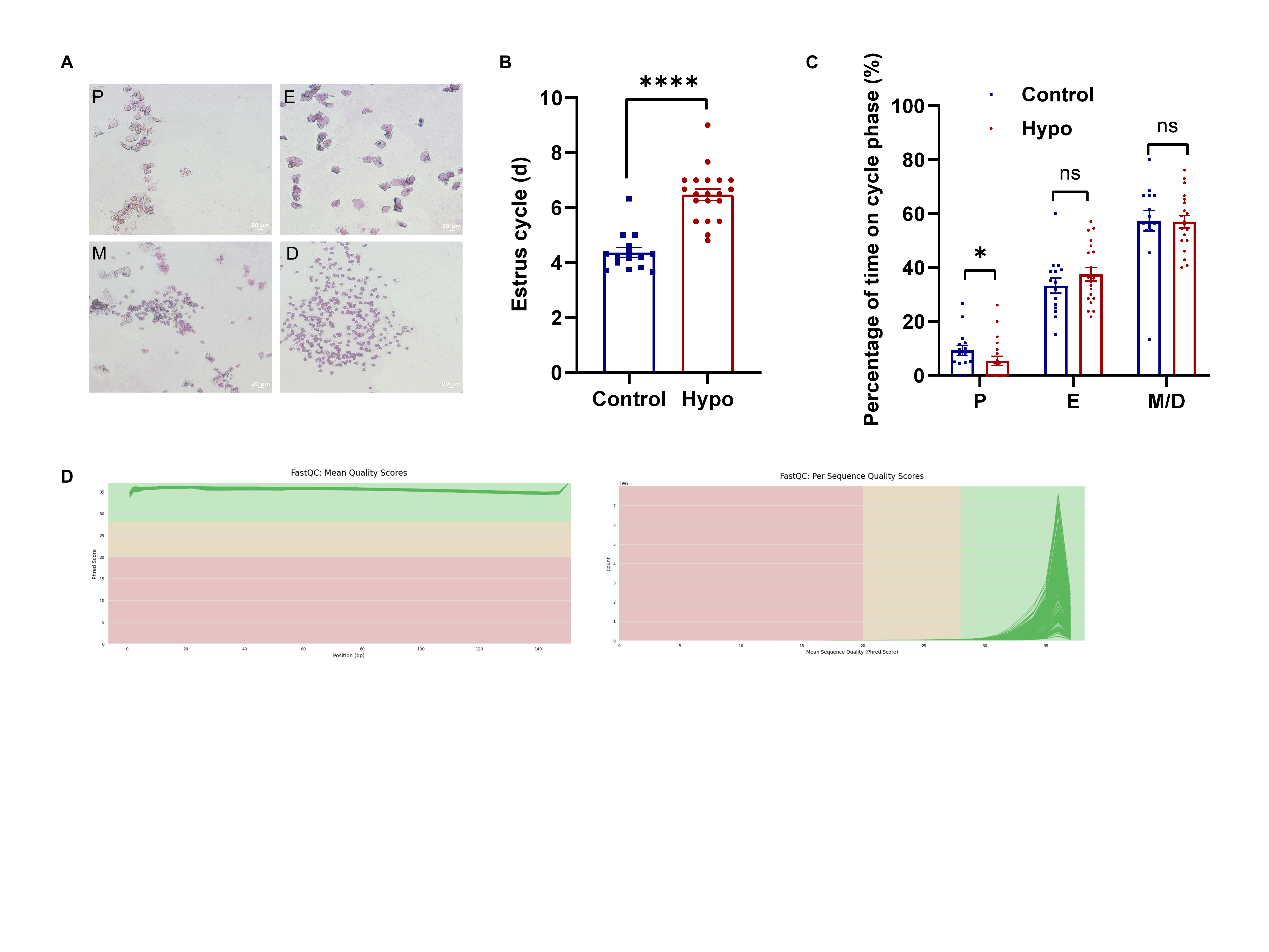


**Supplemental Figure 1.** (A) Representative images of typical vaginal smears at different estrus stages (Wright-Giemsa Staining). (B) Duration of estrus cycle in control and hypo group. Data are presented as Mean ± SEM (n=15 for the control group, n=20 for the hypo group), *****p* < 0.0001. (C) Proportion of each estrus stage. Data are presented as Mean ± SEM (n=15 for the control group, n=20 for the hypo group), *^ns^p* > 0.05, **p* < 0.05. (D) Quality control results of single-cell RNA sequencing data.

**Supplemental Table 1**. Primer sequences of genes for qPCR

| **Gene** | **Forward** | **Reverse** |
| --- | --- | --- |
| *Bcl2* | TCATGTGTGTGGAGAGCGTC | AGTTCCACAAAGGCATCCCAG |
| *Bcl2l1* | CGCCGGAGATAGATTTGAATAACC | CCCGGTTGCTCTGAGACATT |
| *Bad* | ATAACAGTCATCATGGAGGCGCT | CCTTCATCTTCCTCAGTCCCC |
| *Casp3* | GGAGCTTGGAACGCGAAGAA | TTGCGAGCTGACATTCCAGT |
| *Casp9* | TATCATCCCCACCCTCACTCT | GACTTGATCAGTGGGGACTCTG |
| *Gpx4* | TGGATAAGTACAGGGGTTGCG | GTGGGCATCGTCCCCATTTA |
| *Slc7a11* | AACTGCTGGTAATACGCCCC | GGAAAATCTGGATCCGGGCA |
| *Acsl4* | TGGGCTGACAGAATCATGCG | AACTGTATAACCACCTTCCTGC |
| *Lpcat3* | TAAGATGGCGTCTACAGCGG | GTAGAACTGGTGGCCGAAGT |
| *Aifm2* | TAGAGGGTGGTGAGGCCTTG | GTTGGCAGGACACCTCGTTA |
| *Gch1* | CCATTTGTGGGAAGGGTCCA | GGCCACTGCAATCTGTTTGG |
| *Gsdmd* | CTCCCTGGCCCTATTGTCA | GTCTTGCTGCATTCTAACCCTG |
| *Gsdme* | TGCCATTTGCTCCATGCTCTGTC | CGATGCCAAACCTCTCTGTGTCTC |
| *Casp1* | GACTCTAGACTACAGATGCCAAC | CTTCTTATTGGCATGATTCCC |
| *Casp11* | AGACCAATGGCCGTACTCGAA | TGCTGTCTGATGTTTGGTGCT |

**Supplemental Table 2**. Serum levels of TSH, fT3, and fT4 in the control and hypo groups.

|  | Control group  (n=5) | Hypo group  (n=4) | *P* value |
| --- | --- | --- | --- |
| TSH (mU/L) | 3.62±0.21 | 7.46±0.27 | 0.0000 |
| fT3 (pmol/L) | 2.20±0.10 | 0.93±0.03 | 0.0000 |
| fT4 (pmol/L) | 5.30±0.11 | 3.62±0.16 | 0.0000 |

Data are presented as Mean ± SEM (n=5 for the control group, n=4 for the hypo group).

**Supplemental Table 3**. Serum levels of FSH, LH, PRL, E2, and P in the control and hypo groups.

|  | Control group  (n=5) | Hypo group  (n=4) | *P* value |
| --- | --- | --- | --- |
| FSH (mIU/mL) | 5.43±0.18 | 7.98±0.19 | 0.0000 |
| LH (mIU/mL) | 6.59±0.14 | 9.17±0.17 | 0.0000 |
| PRL (ng/mL) | 5.14±0.09 | 7.32±0.25 | 0.0000 |
| E2 (pmol/L) | 47.36±1.83 | 38.93±0.99 | 0.0009 |
| P (ng/mL) | 5.26±0.18 | 3.82±0.13 | 0.0000 |

Data are presented as Mean ± SEM (n=5 for the control group, n=4 for the hypo group).
